# Phenotyping and Exploitation of Kompetitive Allele-Specific PCR Assays for Genes Underpinning Leaf Rust Resistance in New Spring Wheat Mutant Lines

**DOI:** 10.3390/cimb46010045

**Published:** 2024-01-12

**Authors:** Saule Kenzhebayeva, Shynarbek Mazkirat, Sabina Shoinbekova, Saule Atabayeva, Alfia Abekova, Nargul Omirbekova, Gulina Doktyrbay, Saltant Asrandina, Dinara Zharassova, Aigul Amirova, Albrecht Serfling

**Affiliations:** 1 Faculty of Biology and Biotechnology, Al-Farabi Kazakh National University, Almaty 050040, Kazakhstan; sshoinbekova@mail.ru (S.S.); sauleat@yandex.ru (S.A.); nariko21@mail.ru (N.O.); gulina.kaznu@gmail.com (G.D.); saltanat.asrandina@kaznu.kz (S.A.); aigul_amir@mail.ru (A.A.); 2 Kazakh Research Institute of Agriculture and Plant Growing, Almaty Region, Almalybak 040909, Kazakhstan; shynarbek.mazkirat@gmail.com (S.M.); aabekova@mail.ru (A.A.); 3 Mangyshlak Experimental Botanical Garden, Ministry of Science and Higher Education of the Republic of Kazakhstan, Aktau R00A3E0, Kazakhstan; dynara_zharassova@mail.ru; 4 Institute for Resistance Research and Stress Tolerance, Julius Kuehn-Institute (JKI) Federal Research Centre for Cultivated Plants, 06484 Quedlinburg, Germany; albrecht.serfling@julius-kuehn.de

**Keywords:** leaf rust resistance, spring wheat mutant lines, hyperspectral image system, KASP marker

## Abstract

Leaf rust (*Puccinia triticina* Eriks) is a wheat disease causing substantial yield losses in wheat production globally. The identification of genetic resources with permanently effective resistance genes and the generation of mutant lines showing increased levels of resistance allow the efficient incorporation of these target genes into germplasm pools by marker-assisted breeding. In this study, new mutant (M_3_ generation) lines generated from the rust-resistant variety Kazakhstanskaya-19 were developed using gamma-induced mutagenesis through 300-, 350-, and 400-Gy doses. In field trials after leaf rust inoculation, 75 mutant lines showed adult plant resistance. These lines were evaluated for resistance at the seedling stage via microscopy in greenhouse experiments. Most of these lines (89.33%) were characterized as resistant at both developmental stages. Hyperspectral imaging analysis indicated that infected leaves of wheat genotypes showed increased relative reflectance in visible and near-infrared light compared to the non-infected genotypes, with peak means at 462 and 644 nm, and 1936 and 2392 nm, respectively. Five spectral indexes, including red edge normalized difference vegetation index (RNDVI), structure-insensitive pigment index (SIPI), ratio vegetation index (RVSI), water index (WI), and normalized difference water index (NDWI), demonstrated significant potential for determining disease severity at the seedling stage. The most significant differences in reflectance between susceptible and resistant mutant lines appeared at 694.57 and 987.51 nm. The mutant lines developed were also used for the development and validation of KASP markers for leaf rust resistance genes *Lr1*, *Lr2a*, *Lr3*, *Lr9*, *Lr10*, and *Lr17*. The mutant lines had high frequencies of “*a*” resistance alleles (0.88) in all six *Lr* genes, which were significantly associated with seedling resistance and suggest the potential of favorable haplotype introgression through functional markers. Nine mutant lines characterized by the presence of “*b*” alleles in *Lr9* and *Lr10*—except for one line with allele “*a*” in *Lr9* and three mutant lines with allele “*a*” in *Lr10*—showed the progressive development of fungal haustorial mother cells 72 h after inoculation. One line from 300-Gy-dosed mutant germplasm with “*b*” alleles in *Lr1*, *Lr2a*, *Lr10*, and *Lr17* and “*a*” alleles in *Lr3* and *Lr9* was characterized as resistant based on the low number of haustorial mother cells, suggesting the contribution of the “*a*” alleles of *Lr3* and *Lr9*.

## 1. Introduction

Wheat (*Triticum aestivum* L.) is the main food crop grown globally, including in Kazakhstan, for human consumption and for livestock. In many of the world’s wheat-growing areas, fungal diseases significantly restrict its production. Leaf rust (LR) caused by the fungal pathogen *Puccinia triticina* Eriks is one of the most important diseases of wheat worldwide due to its frequent and widespread occurrence, with consequent increases in annual yield losses [1,2,3,4]. This fungal disease also has a significantly negative impact on grain quality depending on the resistance genes present in cultivated varieties [5,6].

Plant resistance to rust diseases can be either quantitative and effective—as in adult plant resistance (APR), which is generally described as horizontal and not vulnerable to being broken down by virulent races of leaf rust—or qualitative, as in seedling resistance (SR), described as vertical and shows a race-specific reaction based on gene–gene interactions [7]. The APR—or partial, polygenic, or slow rusting resistance—caused by minor genes is mainly race-nonspecific but can also be race-specific and short-lived in the case of a single APR gene, being effective only against LR isolates carrying a corresponding avirulence gene. SR, which is conferred by single or major genes, is associated with a programmed cell death defense response known as hypersensitive immunity. Such race-specific resistance confers complete resistance and is highly effective throughout the entire life cycle of the host plants. The APR genes encode different proteins, such as ABC transporters, protein kinases, or hexose transporters [1,8]. Generally, SR genes are qualitatively effective compared to APR genes but lose their effectiveness after several years of utilization [1]. In contrast, durable resistance can be achieved by pyramiding either APR or SR genes or by stacking several of these genes [1,9]. 

The development of cultivars with accurate and correctly combined resistance genes for the desired mode of activity would enable durable resistance and is a long-term breeding program goal. This strategy provides a complex of resistance genes against the dynamics of pathogen virulence and should facilitate lasting resistance (durability). Multiple LR resistance genes (*Lr* genes) of SR and APR have been introduced into wheat cultivars [10]. This is an environmentally friendly and economical way to minimize the negative impacts of LR [1,11]. To date, about 80 genes for LR resistance have been reported [12,13,14] and widely deployed in wheat cultivars. Many of them (44%) are from wild progenitor and non-progenitor species [15] that are often linked to genes conferring undesirable traits [16]. Most of the named genes are associated with SR, conferring race-specific resistance against *P. triticina.* Hence, *Lr* genes, carried by many cultivars (e.g., *Lr3*, *Lr10*, *Lr13*, *Lr17b*, *Lr26*, and *Lr37*), have been overcome [17,18,19], and very few are still broadly effective currently. Only a small portion of them are APR genes [20], including *Lr12*, *Lr13*, *Lr22a*, *Lr22b*, *Lr34*, *Lr35*, *Lr37*, *Lr46*, *Lr48*, *Lr49*, *Lr67*, and *Lr68*, and few of these demonstrate slow rusting resistance. The *Lr* genes, such as *Lr1*, *Lr3*, *Lr10*, *Lr13*, *Lr14a*, *Lr24*, *Lr26*, and *Lr37*, are the most common genes effective against LR [20,21]. 

Mutagenesis is a strong approach for generating novel genetic variation in cereal crops that have limited genetic variability and has been mainly applied to increase yields. However, it has not been extensively used in wheat breeding to achieve durable rust resistance [22,23]. It has been reported that new wheat mutants created by EMS-induced mutagenesis, MNR220, showed enhanced resistance to rust and powdery mildew [24]. Other fast-neutron-derived mutants showed resistance to multiple rust pathogens [25], and a space-induced wheat mutant line (R39) showed APR to stripe rust [5]. The great advantage of mutagenesis is the multi-generation of new advanced mutated alleles of genes that do not exist in a grade germplasm pool and the generation of desired mutations that can be directly used to develop a mutant variety in a short breeding cycle [26,27]. Mutant resources of crops are not considered to be genetically modified organisms (GMOs) and are, therefore, more acceptable to the public. 

Our research on developing spring wheat mutant lines (M_3_–M_7_ generation) based on cv. Almaken, Zhenis, and Eritrospermum-35 to improve genetic variability showed these mutants’ higher productivity and grain morphometry along with bio-fortification ability [28,29,30,31]. In parallel, mutant lines with increased grain, Fe and Zn content revealed organ-specific gene expression profiles of genes involved in intracellular iron transport, providing new insights into iron uptake, translocation rate, storage, and regulation in wheat and aiding the prioritization of gene targets for Fe bio-fortification and bioavailability [30]. We also revealed an Fe-deficiency response in a highly differential expression of iron homeostasis-related genes in spring wheat mutant lines with increased grain Fe content [31]. 

High-throughput phenotyping for disease resistance based on hyperspectral image system (HSI) analysis techniques has rapidly increased in recent years, providing the non-destructive detection and classification of plant diseases and stress [32,33,34,35,36]. The HSI is an ideal tool for capturing biophysical variations caused by crop infestations due to its narrow bands and high spectral resolution. Hyperspectral reflectance has previously been used to measure biochemical and physiological traits in wheat [37]. 

Genetic diversity is an effective way to maintain the sustainable rust resistance of wheat. Kompetitive allele-specific PCR (KASP) is a uniplex and flexible genotyping platform that is time- and cost-effective and suitable for the high-throughput genotyping of SNPs or inserts and deletions (InDels) [38]. In wheat, KASP markers have been developed and validated for rust resistance [39], wheat streak mosaic virus resistance [40], and fusarium head blight resistance gene *Fhb1* [41]. This allows the identification of desirable germplasm for breeding and the development of strategies for building a pyramid of superior genetic variation for target traits [1]. KASP assays have been developed for *Lr14a*, *Lr16*, *Lr21*, *Lr22a*, *Lr23*, *Lr27* (*Sr2*), *Lr34*, *Lr37* (*YR17*), *Lr67*, *Lr68*, *Lr78*, and *LrCen* [42,43,44,45,46,47,48]. There have been no reports on the development and exploitation of KASP assays for *Lr1*, *Lr2a*, *Lr3*, *Lr9*, *Lr10*, and *Lr17*, and there is thus a need to validate gene-specific KASP markers for genes underpinning LR resistance in new spring wheat mutant lines.

The objectives of the present study were (1) to broaden the genetic variability of spring bread wheat on the basis of rust-resistant cv. Kazakhstanskaya-19 via mutation breeding and to develop new M_3_ mutant lines through gamma irradiation doses of 300, 350, and 400 Gy; (2) to carry out APR phenotyping of mutant lines in a rust-infection field trial; (3) to evaluate mutant lines identified as APR to LR for their SR by microscopic observation and HIS analysis; and (4) to develop and exploit KASP assays for LR resistance genes *Lr1*, *Lr2a*, *Lr3*, *Lr9*, *Lr10*, and *Lr17*.

## 2. Materials and Methods

### 2.1. Plant Materials, Application of Induced Mutagenesis, and Phenotypic Evaluation 

To develop mutant lines (M_3_ generation), spring wheat (*Triticum aestivum* L.) grains of the rust-resistant cultivar Kazakhstanskaya-19 (released and cultivated in Kazakhstan) were treated with 300-, 350-, and 400-Gy irradiation doses from a ^60^Co research irradiator located at the plant breeding and genetics laboratory of the IAEA laboratories in Seibersdorf, Austria. These three irradiation doses were selected based on an analysis of radiosensitivity (carried out at this laboratory) in which LD_50_ was found to be 330 Gy for cv. Kazakhstanskaya-19. Initially, to generate these mutant lines, 3000 grains of the variety for each dose were irradiated. The grains were planted after irradiation treatment to raise M_2_ plants. The M_2_ generation was grown in the experimental field of the Kazakh Research Institute of Agriculture and Plant Growing (Almaty region, Kazakhstan; 43°15′ N, 76°54′ E, elevation 550 m above mean sea level). Advanced single spikes were selected from individual plants for the development of M_2_ plants. Later, the grains were preferentially picked from the main spike of the best-yielding mutant lines (M_2_ generation). Each M_3_ mutant plant produced only a single main spike. The selection criteria for the lines included the mean of grain weight per main spike based on the values for the parent, cv. Kazakhstanskaya-19. Grain samples from each mutant population were planted together with cv. Kazakhstanskaya-19 in the infectious field and used for further evaluation of the response to LR during the growth season. In order to derive KASP markers, near-isogenic lines (NILs) carrying *Lr* genes were obtained from different genebanks of the Leibniz-Institut für Pflanzengenetik und Kulturpflanzenforschung (IPK, Gatersleben, Germany) and CIMMYT (El Batán, Mexico).

### 2.2. Evaluation of Adult Plant Resistance to LR in an Infectious Experimental Field

Inoculation with LR-uredospore water suspension containing 0.001% Tween-20 was carried out twice at the booting stage, with an interval between inoculations of 10 and 12 days. Plants were treated after preliminary moistening in the evening, and conditions of high humidity were maintained. The infectious material was the spore inoculum of the fungus *P. triticina* of the Kazakh rust population, provided by the Scientific Research Institute of Biological Safety Problems of the Ministry of Education and Science of the Republic of Kazakhstan. Infection type was recorded on flag leaves in late May and early June, when plots were at boot and milk stages, respectively. For the accumulation and spreading of infection in the nursery, a susceptible cultivar, “Morocco”, was grown between experimental plots. Scoring of LR symptoms was performed according to the method developed at the CIMMYT [49]. The five infection types were 0—immune, R—resistant, MR—moderately resistant, MS—moderately susceptible, and S—susceptible, comparable to other scoring systems available in [50,51,52,53] (Supplement S1). The time of the second evaluation was also determined when rust severity in the susceptible control Morocco reached an infection level of 60–80% of the leaf area. A total of 75 immune or resistant M_3_ mutant lines were selected together with cv. Kazakhstanskaya-19 (wild type) for the subsequent experiments. The mutant germplasm included 42 genotypes of 300-Gy-dosed, 16 lines of 350-Gy-dosed, and 17 lines of 400-Gy-dosed grains. Cv. Kazakhstanskaya-19 was recorded as moderately LR resistant. 

### 2.3. Rust Isolates for Evaluating Seedling Leaf Rust Resistance 

All isolates of leaf rust were used for the SR test and initially derived from single pustules on the susceptible genotype “Borenos”. Races of virulent *P. triticina* isolates (PT: “4083”, “4171”, “77WxR”, “FI17”, “HkLr13”) were determined according to McIntosh et al. [51]. *Lr12*, *Lr13*, *Lr22*, *Lr35*, *Lr37*, and *Lr46* are race-specific APR genes [1, 52, 53] carried by NILs and have been included in the virulence analysis according to Qureshi et al. [25]. Virulence against all of these *Lr* genes was observed at the seedling stage (Table 1). 

### 2.4. Evaluating LR Resistance at the Seedling Stage in Greenhouse Experiments

Seedling assays were performed with cv. Kazakhstanskaya-19 and a panel of leaf- and yellow rust mutant lines previously phenotyped as ADP resistant and selected in replicated semi-controlled greenhouse experiments at the Institute for Resistance Research and Stress Tolerance, Quedlinburg, Germany, using detached leaf assays of 10-day-old seedlings [11]. Three seedlings of each line, each with two replicates per isolate, were grown in 7 × 11 potting trays filled with soil substrate Fruhstorfer Typ T (Hawita Gruppe GmbH, Vechta, Germany) and raised at 21 °C until the second leaf was fully expanded. Ten-day-old seedlings were treated by spraying with 0.005% aqueous Tween^®^20 (Carl Roth GmhH+Co.KG, Karlsruhe, Germany) to facilitate the smooth adhesion of spores to the leaves. Inoculation was performed with 30 mg of uredospores of each isolate mixed with clay (1:2) using a powder atomizer. Subsequently, the 10-day-old seedlings were inoculated with *Puccinia triticina 77WxR* isolate. To maximize infection, seedlings were then grown at 10–12 °C for PS and at 18–20 °C for PT at a photoperiod of 14/10 h with supplementary lighting. Leaf samples were taken at 72 and 168 h after inoculation (hai) and stained using Calcofluor White to calculate haustorial mother cells (HMC). The susceptible standard variety Borenos was used as a control. Inoculation was implemented in an air-blowing inoculation tower using uredospores of the aggressive LR isolate *77WxR* (Table 1) [3,53].

### 2.5. Staining Procedures and Microscopy

In order to count the formation of fungal structures (HMCs, haustoria) after inoculation of isolate *77WxR*, microscopic observation was used. Leaves of inoculated plants were collected directly before inoculation (0 h) and at 72 and 168 h by cutting leaves into segments of 2 cm length. Three leaf segments from the middle of the third youngest leaf of three seedlings of the same variant were analyzed. Within each replication, 15 infection sites (germinated uredospore and appressorium generated) were counted so that, altogether, counts of 30 infection sites (3 × 10) were used for the assessment. Fungal cell walls were stained using Calcofluor White M2R solution (0.2% in sterile water, *w*/*v*) [54] optimized for the staining of leaves for 10 min of incubation at room temperature. Samples were washed four times with sterile water, transferred to a microscope slide, and embedded in a glycerol/water solution (1:1 *v*/*v*). Microscopy of leaf cells and fungal structures was performed using an Axioskop 50, while an Axiocam MRc connected with the software package Axiovision 4 (Carl Zeiss AG, Jena, Germany) was used for taking pictures and for the analyses. The Calcofluor White M2R-stained fungal structures were observed using filter set 02 (excitation filter G 365, beam splitter FT 395, and barrier filter LP 420), and autofluorescence within plant tissue was recorded using filter set 05 (excitation filter BP 400–440, beam splitter FT 460, barrier filter LP 470) according to Serfling et al. [11].

### 2.6. Canopy-Scale Hyperspectral Imaging Measurement and Data Acquisition

A line-scanning HSI system covering the visible and near-infrared (NIR) wavelengths (404–2511 nm with 3.2 nm spectral resolution) was used in this study. For the assessment of the 14-day effect of LR infection on leaves, canopy percentage reflectance data were acquired with two cameras: one visible-NIR camera for spectral sampling from 400 to 1000 nm and 16-bit resolution and one short wave IR camera with a spectral range from 930–2500 nm, both with 16-bit resolution (Hyspex, Neo, Oslo, Norway). Cameras together with a continuous light halogen lamp (C12, Hedler Systemlicht, Limburg, Germany) were mounted on a linear unit (Standa, Vilnius, Lithuania) of 1 m length in a room protected from external light. The cameras and linear unit were coordinated using Hyspex Ground (ver. 3.5, Norsk Elektro Optikk, Oslo, Norway), and the conversion of images to spectral radiance was conducted using Hyspex RAD software (ver. 3.5, Norsk Elektro Optikk, Oslo, Norway). The HSI was taken using a fixed 30 cm lens. The spectrometer had a sampling interval of 1.4 nm for the 350–1050 nm region of the electromagnetic spectrum (3 nm spectral resolution) and 2 nm for the 1050–2500 nm region (10 nm spectral resolution), with a field of view of 25°. Both 1.4 nm and 2 nm sampling intervals were automatically interpolated to 1 nm intervals by the instrument. The sensor, facing downwards at the center of the plot, was positioned 0.5 m from the top of the wheat canopy, covering a 22.16 cm diameter field of view. The instrument was referenced to a calibrated spectral on a white reflectance panel about every 15 min while readings were obtained, allowing readings from different assessment dates to be compared. All samples were placed on a blackboard with low reflectivity, and both ends were fixed with non-reflective tape to reduce the influence of unevenness. Before inoculation, all samples were scanned as healthy samples.

Standardized imaging of infected leaf assays was further realized by the automated phenotyping platform Macrobot, while BluVision software (“BluVision Macro” described by Lueck et al., 2020) was used to analyze the images. Briefly, the software detects leaf samples and uredospore pustules based on color and measures their pixel sizes. The relation between leaf size and pustule size results in the rated percentage of infected leaf area [55]. A more detailed description of the experimental setup can be found elsewhere [56]. We examined and scored the 14-day influence of LR resistance on 75 genotypes. All genotypes were grown under greenhouse conditions for 5 (BBCH19; T2) and 10 weeks (BBCH25; T3), each in 7 replications. Subsequently, resistance screening was undertaken using detached leaf assays and the above-described procedure. Pictures were taken separately as red, green, and gray-scaled files using an RGB camera. BluVision software was used to calculate the leaf area and the infected leaf area based on pixel codes defined for uredospore colonies of leaf rust. This technique was used to validate the hyperspectral analysis data. The list of spectral indexes used for detection of 14-day LR infection in Kazakhstanskaya-19 mutant spring wheat lines at the leaf- and canopy scale is shown in Table 2.

### 2.7. DNA Extraction and KASP Primer Design and Validation

Genomic DNA of cv. Kazakhstanskaya-19 and 74 mutant lines were extracted from leaves of 4-day seedlings according to the Miniprep method described by [65]. Extraction of genomic DNA was performed using CTAB (cetyltrimethylammonium bromide) buffer and 2-Mercaptoethanol and is described in Supplement S1. After solution of the DNA pellet in TE (tris(hydroxymethyl)aminomethane (TRIS) and ethylenediaminetetraacetic acid (EDTA) and overnight incubation at 4 °C as a last step, DNA concentration was measured using NanoDrop 1000 (ThermoFisher Scientific, Martinsried, Germany). *Lr*-specific KASP markers were also used to detect *Lr* genes in the wild-type and mutant lines. PCR analysis using KASP-markers was performed in a real-time PCR system (ABI7500 fast, ThermoFisher Scientific, Germany) using the following thermal cycling conditions: hot start at 94 °C for 10 min, followed by 10 touchdown cycles (95 °C for 20 s; touchdown 65 °C, 1 °C per cycle, 25 s) and then 30 cycles of amplification (95 °C for 10 s; 57 °C for 60 s). KASP assays were run with 10 μL final reaction volumes, containing 5 μL KASP master mix (BioSearch Technologies, Teddington, Middlesex, UK) following the instructions available on the website https://www.biosearchtech.com/products (accessed on 17 December 2023). Primer mix (0.14 μL), 2 μL of 10–20 ng/μL genomic DNA, and 2.86 μL of water were added. KASP marker analysis included the design and development of KASP markers for specific rust diseases: *Lr1*, *Lr2a*, *Lr3*, *Lr9*, *Lr10*, *Lr17*, *Lr14*, *L19*, and *Lr24*. Sequences of allele-specific and common primers are listed in Table 2. KASP primers were designed based on sequence information from https://www.cerealsdb.uk.net/cerealgenomics/CerealsDB (accessed on 17 December 2023) and the tool available for KASP primer selection [66].

#### Identification of Chromosomal Regions Consisting of Lr Genes in NILs

NILs carrying *Lr1*, *Lr2a*, *Lr3*, *Lr9*, *Lr10*, *Lr17*, *Lr14*, *Lr19*, and *Lr24*, and the susceptible cultivars “Thatcher” (recurrent parental line) and “Monopol” were analyzed using the 90K Illumina Infinium Chip for wheat (TraitGenetics GmbH, Gatersleben, Germany). Out of 81,587 SNPs from the array, a subset of uniquely mapped ones from the physical consensus map of [67] was used, i.e., a total of 51,680 SNP markers. Alleles from selected SNP markers were ordered based on physical positions and chromosomes. The search was restricted to the chromosome to which the *Lr* gene was previously assigned by McIntosh et al. [51]. Sequences from genes that have been reported as isolated were downloaded from the https://www.ncbi.nlm.nih.gov/nuccore website (accessed on 17 December 2023). In order to identify the physical region of known *Lr* genes, sequence information was blasted to the available reference genome [68] (BLAST-WHEAT URGI (inra.fr)). Physical regions were then compared to the physical marker positions. SNP markers showing different alleles between recurrent parental line Thatcher and all other NILs were used for the derivation of KASPs. Heterozygous signals in recurrent parent Thatcher or a NIL were excluded from further analysis.

### 2.8. Statistical Analysis

Statistical analysis, including one-way ANOVA, SD, SEM, and *p*-value, was carried out using R-Studio (Version 1.1.456) and JMP17 (SAS Institute GmbH, Heidelberg, Germany). All values were expressed as the mean of three measurements (biological replicates) for each gene.

## 3. Results

### 3.1. Microscopic Evaluation of Haustorial Mother Cells of LR Isolate in Near-Isogenic Lines (NILs) Carrying LR Resistance and Spring Wheat Mutant Lines

**Figure 1:**
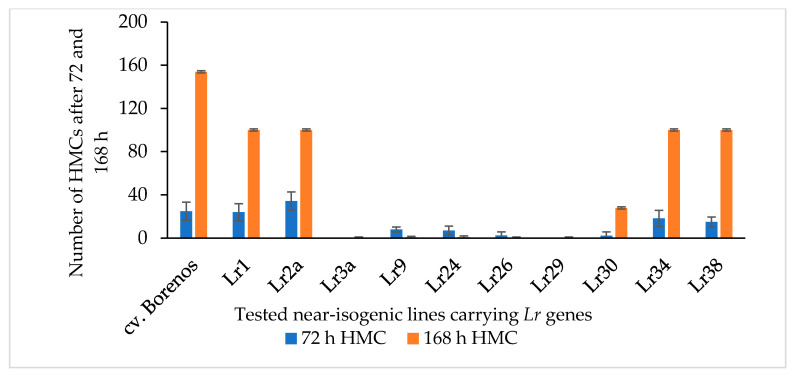
Number of haustorial mother cells (HMCs) produced 72 and 168 h after inoculation with *P. triticina* fungal isolate *77WxR* in 10-day-old seedlings of the LR-susceptible standard cv. Borenos (control), *Lr1*, *Lr2a*, *Lr3a*, *Lr9*, *Lr24*, *Lr26*, *Lr29*, *Lr30*, *Lr34*, and *Lr38*. These *Lr* genes are race-specific APR genes carried by near-isogenic lines (NILs). Values are the mean ± standard deviation of 15 infected leaf area sites.

To expand the known LR-resistant genetic resources in spring wheat by identifying new mutants with SR and APR, new mutant lines (M_3_ generation) were developed using the rust-resistant cv. Kazakhstanskaya-19 wild parent (WP) through 300-, 350-, and 400-Gy irradiation. These differently dosed mutant lines, along with cv. Kazakhstanskaya-19, were evaluated in a rust-infected field trial in the Almaty region of Kazakhstan. Mutant plants without any visible symptoms of disease manifestation, according to [50,51], were selected and recorded as APR (Supplement S1). The *P. triticina* fungal isolate *77WxR* has previously been reported to produce the highest number of uredospores among the known isolates [11]. In the present study, *77WxR* was included in the virulence analysis for the SR evaluation. The 300-, 350-, and 400-Gy-dosed spring wheat mutant lines (a total of 75 samples) were utilized for counting the formation of fungal haustorial mother cells (HMCs) at 72 and 168 hai with *77WxR* on leaves of 10-day-old seedlings via microscopy. The cv. Borenos—an LR-susceptible genotype (disease score 4)—was used as a control. Additionally, 10 NILs carrying race-specific APR *Lr* genes located on the A-genome, such as *Lr1*, *Lr2a*, *Lr3a*, *Lr9*, *Lr24*, *Lr26*, *Lr29*, *Lr30*, *Lr34*, and *Lr38*, were included in the same assessments (Figure 1). Except for the *Lr3a*, *Lr26*, and *Lr29* lines, which had no visible HMCs 72 and 168 hai with *77WxR*, the isolate was virulent against all tested *Lr* genes of the NIS lines, with a progressively increasing number of HMCs (Figure 1). The highest time-dependent *77WxR* virulence effect was observed on LR-susceptible Borenos and, to a lesser extent, in the *Lr1*, *Lr2a*, *Lr34*, and *Lr38* NIS lines (Figure 1).

**Figure 2:**
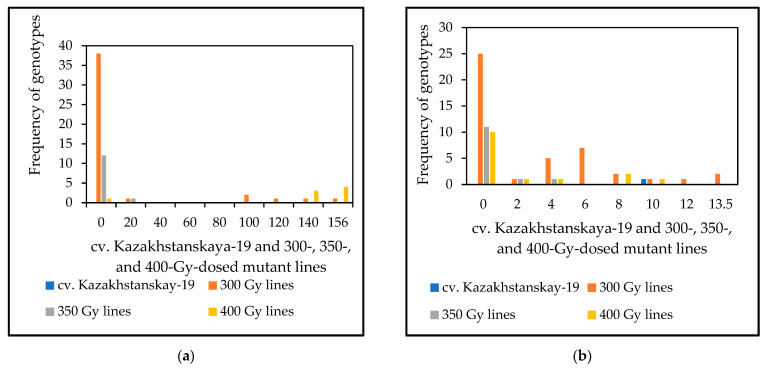
Frequency distribution of genotypes in haustorial mother cells (HMCs) produced 72 (**a**) and 168 (**b**) hours after inoculation with *77WxR P. triticina* in 10-day-old seedlings of M_3_ spring wheat mutant lines generated using cv. Kazakhstanskaya-19 through 300-, 350-, and 400-Gy irradiation. Values are the mean of 15 infected leaf area sites ± standard deviation. A list of spring wheat genotypes is presented in Supplement S1.

The results of the microscopy evaluation of HMC production 72 and 168 hai with *P. triticina* fungal isolate *77WxR* in Kazakhstanskaya-19 mutant lines generated through different doses of gamma irradiation are shown in Figure 2 and Supplement S1. From 42, 15, and 17 samples representing the 300-, 350-, and 400-Gy-dosed mutant lines, respectively, and having APR, we identified 37 (88.1%), 12 (80%), and 14 (82.4%) lines, respectively, as SR at both times following fungal inoculation. Thus, for HMC formation, the results indicated that most of the mutant lines identified as APR to LR had a strong association with SR.

### 3.2. Microscopic Observation of the Development of Fungal Structures as a Time-Dependent Inoculation Response to P. triticina Fungal Isolate 77WxR in Different Spring Wheat Genotypes

**Figure 3:**
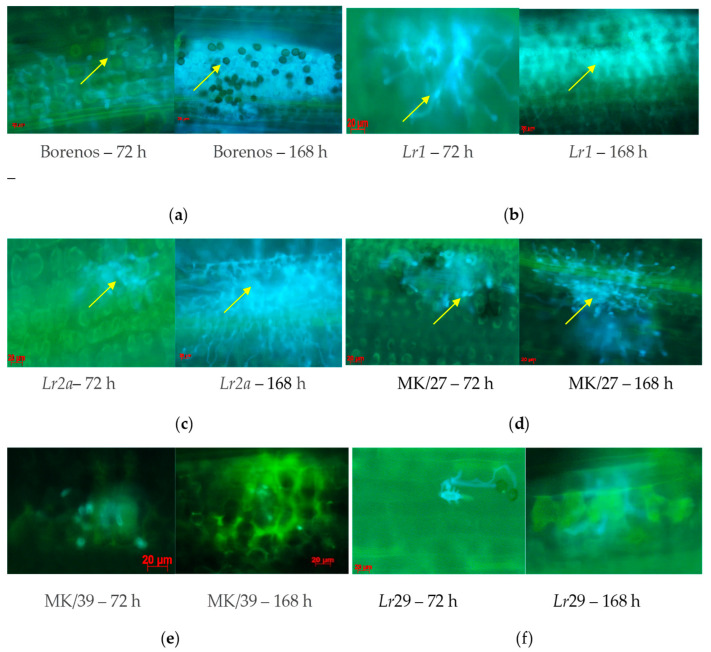
Microscopic signs of fungal structure development on leaf segments in LR-susceptible seedling (SS) genotypes Borenos (**a**), SS *Lr1* (**b**), and SS *Lr2a* (**c**), spring wheat Kazakhstanskaya-19 SS mutant line MK/27 (**d**), SR Kazakhstanskaya-19 line MK/39 (**e**), and SR *Lr29* (**f**) NIL lines at 72 and 168 h after inoculation with *P*. *triticina* isolate *77WxR*. Yellow arrows—pustules with blue balls or dots.

The development of fungal structures was observed by microscopy as a time-dependent response 72 and 168 hai with *77WxR* on the LR-susceptible cv. Borenos, and race-specific NIL *Lr1*, *Lr2a*, *Lr3a*, *Lr9*, *Lr24*, *Lr26*, *Lr29*, *Lr30*, *Lr34*, and *Lr38* (Figure 3). This fungal isolate was also used to monitor the responses of incompatible and compatible resistance mechanisms in SS Kazakhstanskaya-19 MK/27 and SR mutant lines (MK/39) selected based on their significantly different HMC counts after both inoculation periods (Supplement S1).

In line with the large number of HMCs detected (Figure 1 and Figure 2) at 72 and 168 hai with *77WxR* in SS cv. Borenos (Figure 3a), SS *Lr1* (Figure 3b), SS *Lr2a* (Figure 3c), and SS Kazakhstanskaya-19 350-Gy-dosed MK/27 mutant lines (Figure 3d), clear progressive production of uredospore pustules on leaves was observed as a temporary reaction. Most of the pustules had a particular root-like network with blue balls or dots (indicated by yellow arrows). In the Kazakhstanskaya-19 350-Gy-dosed MK/39 mutant line selected as the SR (Figure 3e and Supplement S1) and in the SR *Lr29* line (Figure 3f), no development of HMCs and uredospore pustules was observed. In addition, autofluorescence as an indicator of plant defense reactions that could prevent the formation of HMCs was most visible at 72 and, to a lesser extent, 168 hai (Figure 3e,f). In accordance with this phenomenon, no HMCs were formed in SR genotypes to LR selected at both infection durations.

### 3.3. Application of Hyperspectral Imaging System for the Quantification of Healthy and Diseased Plants Based on Different Indexes, and Discrimination between Susceptible and LR-Resistant Spring Wheat Mutant Lines

**Figure 4:**
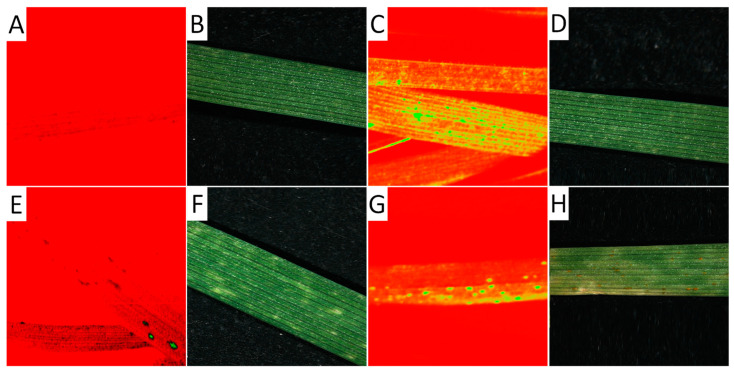
Hyperspectral image of leaves from seedlings 14 days after germination. Hyperspectral images were taken at a wavelength of 694.57 nm (**A**,**C**,**E**,**G**) and separately as RGB images (**B**,**D**,**F**,**H**) of a non-infected leaf (**A**,**B**), an infected leaf from already-developed but not yet macroscopically visible uredospore pustules (**C**,**D**), a leaf showing hypersensitive reaction after an infection (**E**,**F**), and a leaf rust-infected leaf showing uredospore pustules.

HSI analysis of the differences in the reflectance values in the visible range combined with the NIR range is a non-invasive method of characterizing plant-pathogen interactions [32,33,34,36]. In the present study, this tool was used to assess healthy and diseased plants’ responses and discriminate between susceptible and resistant spring wheat mutant lines in 10-day-old seedlings 14 days after fungal inoculation (Figure 4). The hyperspectral data were in the spectrum range of 404–2495 nm, with a spectral resolution of 3.2 nm.

**Figure 5:**
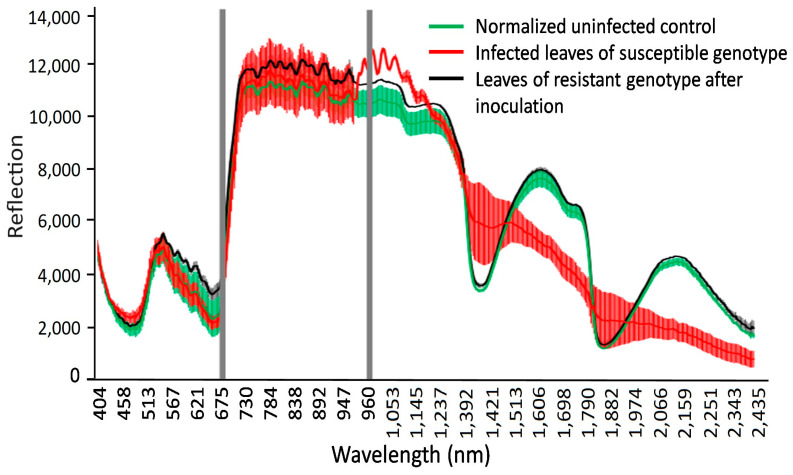
Reflectance of non-(**green**), infected leaves of susceptible genotypes (**red**), and resistant genotypes showing hypersensitive spots (**black**). Gray areas show wavelengths with the most significant differences in reflectance at 694.57 and 987.51 nm.

**Figure 6:**
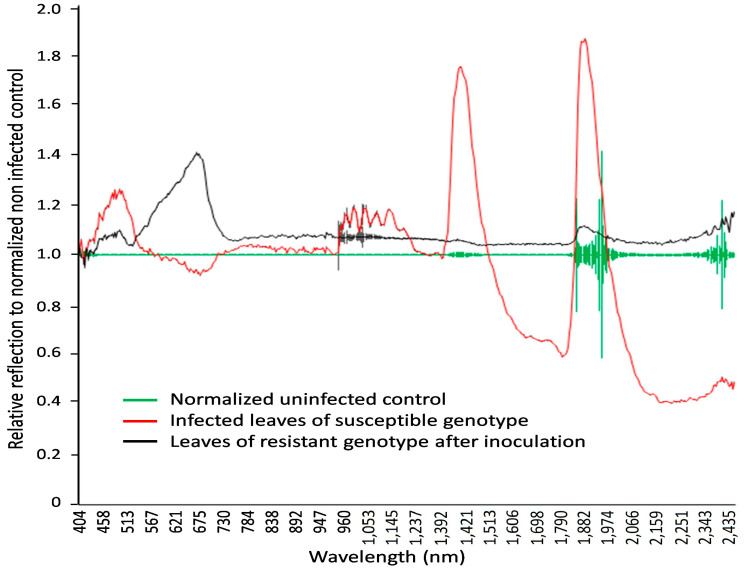
The normalized reflection values in wavelengths from 404 to 2511 nm, with 3 nm spectral resolution, for non-infected and LR-infected seedlings in spring wheat mutant lines two weeks after inoculation. Fifteen samples each were used for non-infected (**green line**), infected (**red line**), and resistant genotypes (**black line**) (hypersensitive spots). Leaves of infected wheat genotypes showed a relative reflectance increase in visible light, with the highest reflectance at 462 nm and 644 nm and peak near-infrared reflectance at 1936 nm and 2392 nm.

The hyperspectral image at a wavelength of 694.57 nm shows the difference between non-infected and infected leaves of wheat seedlings, with fungal structures under the leaf surface and distinct hypersensitive spots and uredospore pustules (Figure 4). It is clearly visible that no—or only unspecific—symptoms are visible in the RGB pictures taken using a conventional camera. In particular, the hypersensitive spots representing a defense reaction against rust are difficult to distinguish from chlorosis in the RGB images (compare Figure 4D,F). This wavelength could be identified as the most different in a comparison of the reflectance of non-infected leaves, infected leaves of susceptible genotypes, and inoculated leaves of resistant genotypes showing some hypersensitive spots (Figure 5). The reflectance values of both variants were then compared to the normalized (reflectance defined as “1”) reflectance values of the non-infected control (Figure 6).

Spectral vegetation indexes utilize the characteristic shape of the green vegetation spectrum by combining the low reflectance (R) of the visible wavelengths with the high R of the near-infrared wavelengths. These indexes are grouped into three categories, one of which is the intrinsic group [18]. In the present study, the red edge NDVI, structure insensitive pigment index (SIPI), and ratio vegetation index (RVSI) were found to be strongly correlated with LR infection (Table 2). Additionally, the spectral indexes related to water index (WI) and normalized difference water index (NDWI) also clearly differentiated the response between non-infected and LR-infected wheat plants (Table 2). The LR disease severity index (LRDSI) [46] was significantly higher (*p* < 0.015) in infected plants than in healthy plants (Table 2).

**Figure 7:**
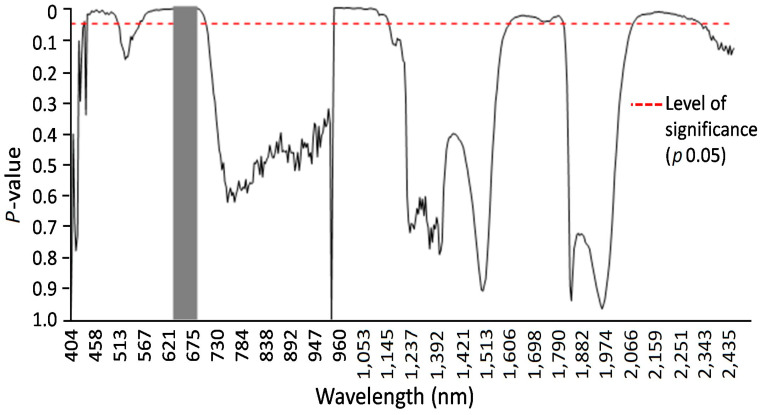
Results of F-tests at alpha 0.05 (marked by the red dashed line) of hyperspectral imaging system analysis for susceptible and resistant spring wheat mutant lines two weeks after LR infection. A Tukey’s test (alpha 0.05) was used to compare averages of reflection values with significant F-test results. Significantly different reflectance values could be identified and are marked by the gray vertical area.

Specific wavelengths indicating changes in leaf spectral properties produced by LR in susceptible and resistant mutant lines could be elucidated using statistical analysis (via ANOVA and Tukey’s test for each individual wavelength) of reflectance from non-infected, susceptible, and resistant genotypes (Figure 6 and Figure 7). A number of spring wheat mutant lines were selected for the evaluation of LR resistance by HSI analysis based on ratings in greenhouse trials (Supplement S1). Qualifying the severity of the disease by analyzing the visible symptoms showed that most of the mutant lines (70 samples) showed SR to LR (Supplement S1). Some mutant lines—numbered as MK/15, MK/17, MK/20, MK/36, MK/37, MK/46, and MK/53—had SR to LR. The lines numbered MK/15, MK/17, MK/20, MK/36, and MK/37 were the Kazakhstanskaya-19 line developed using 400 Gy, MK/46 using 350 Gy, and MK/53 using 300 Gy. The most significant differences between susceptible and resistant mutant lines were found to be reflected at 694.57 and 987.51 nm (Figure 6).

### 3.4. Development and Validation of KASP Markers for LR Resistance Genes

Mutant breeding lines generated using different Gy irradiation levels were studied for SNPs in nine *Lr* genes using KASP markers developed based on the SNP information and the physical positions of markers in the reference genome [59,60,66,68]. From 51,160 mapped markers on chromosomes known to carry *Lr1* (5D), *Lr2a* (2DS), *Lr3* (6B), *Lr9* (6B), *Lr10* (1A), *Lr14a* (7B), *Lr17* (2A), *Lr19* (7D), and *Lr24* (3D), KASP markers could be selected based on allele information for the NILs (Table 3). Three KASP markers for genes *Lr14*, *Lr19*, and *Lr24* were not carried by any genotype, were monomorphic, and were excluded from further analysis. The genotyping results for the remaining six *Lr* genes—*Lr1*, *Lr2a*, *Lr3*, *Lr9*, *Lr10*, and *Lr17*—are presented in Supplement Figure S1 and summarized in Table 4.

Among the 17 mutant lines originating from genotypes developed through 400-Gy irradiation, 14 mutant lines were completely homozygous for the “*a*” SNP alleles in six studied *Lr* genes using KASP markers. All 14 mutant lines showed no symptoms and no fungal HMC development (Supplement Figure S1). In contrast, three mutant lines from the same high-dosage Gy irradiation were identified as homozygotes for “*b*” SNP alleles in five studied *Lr* genes, while genotyping for *Lr3* in the three mutant breeding lines was “undetermined”. Additionally, the “*a*” SNP alleles in KASP markers were found in mutant lines MK/22 and MK/23 for genes *Lr9* and *Lr10*, respectively. Nevertheless, all these three mutant lines showed very high levels of fungal infection and haustoria development (Supplement Figure S1).

A similar pattern of KASP allele distribution was found in the 16 mutant lines originating from a moderate (350-Gy) dosage of irradiation and is presented in Supplement Figure S1. Only one mutant line (MK/41) had “undetermined” status for four genes—*Lr3*, *Lr9*, *Lr10*, and *Lr17*—while the other mutant lines were fully homozygous for SNP genotyping using KASP markers in all studied *Lr* genes. It is important to note that all 13 mutant lines showed resistant phenotypes for haustoria development and fungal infection in leaves (Supplement Figure S1). Among another three mutant lines with homozygous “*bb*” genotypes, only one mutant line (MK/25) had SNP allele “*a*” for the *Lr10* gene, and SNP genotyping for *Lr3* was “undetermined”. For the phenotyping, the fungal infection and haustoria development exhibited were very strong, with high levels in the three mutant lines (Supplement Figure S1).

For the last group of wheat mutant breeding lines, produced via 300-Gy irradiation, 39 homozygous genotypes had allele “*a*” of KASP markers for all six studied *Lr* genes, while only three other mutant lines were identified as “*bb*” genotypes. Among the 39 mutant lines, allele “*b*” was found in *Lr3* in a single line (MK/76), which was very similar to the first two groups of mutant lines described above (Supplement Figure S1). Another mutant line (MK/54) showed high genotypic diversity, being homozygous for the “*a*” allele in the *Lr3* and *Lr9* genes but having “*b*” alleles in four other genes: *Lr1*, *Lr2a*, *Lr10*, and *Lr17*. Nevertheless, these two lines—and other mutant lines classified as “*aa*” genotypes—showed neither infection nor HMCs. The exception was found only in a single mutant line, MK/84, which had moderate susceptibility to the fungus and some haustoria growth (Supplement Figure S1).

The last three mutant lines from Supplement Figure S1, identified as “*bb*” genotypes using KASP markers—showed very different patterns of genotyping and phenotyping. Only one mutant line (MK/28) of the “*bb*” genotype showed high numbers of HMCs. The second mutant line, MK/29, had one SNP with an “*a*” allele in the KASP marker for *Lr10* but also a high level of fungal infection. However, the mutant line MK/30—with “*bb*” genotypes for all six studied *Lr* genes—showed excellent resistance to the fungus, with “zero” haustoria registered (Supplement Figure S1).

## 4. Discussion

### 4.1. Microscopic Evaluation of HMCs of LR in NIL Carrying LR Resistance Genes and Spring Wheat Mutant Lines Generated from cv. Kazakhstanskaya-19 through Different Doses of Gy Irradiation

The continuous identification and selection of rust-resistant genetic resources is an important strategy employed by wheat breeding programs. The identification of resources with effective and durable resistance genes allows the efficient incorporation of these target genes into germplasm pools [1,19]. Breeding efforts have been undertaken to introgress various *Lr* genes into wheat breeding lines [69]. Enhanced host plant resistance—and, more importantly, the combination of several effective *Lr* genes—remains the most feasible, economical, and environmentally friendly approach to ensuring durable resistance [70,71,72,73]. Wheat cultivars and breeding lines with multiple *Lr*-resistant genes have significantly lower disease levels [74,75], and the use of this kind of resistance has the potential to significantly reduce disease epidemics. A significant number of the *Lr* genes are race-specific and generally conform to the “gene-for-gene” model proposed by Flor [76], conferring resistance to pathogen races with corresponding *Avr* genes.

The main goal of this work was to extend the spring wheat genetic variation demanded by the development of new, valuable rust-resistant sources through mutation breeding and to examine the resistance of the resulting mutant lines at two stages of development. Macroscopic and microscopic analyses of infection were combined with genotyping with KASP markers for *Lr* genes, and comparison with phenotyping evaluation through HSI system imaging was used for the detection of healthy and diseased plants and discrimination between susceptible and resistant spring wheat mutant lines. A total of 75 new mutant lines (M_3_ generation), generated from a moderately resistant spring wheat variety (cv. Kazakhstanskaya-19) exposed to 300-, 350-, and 400-Gy irradiation, were selected due to their demonstrated APR resistance to LR in a field infection trial in Kazakhstan (Supplement S1). These Kazakhstanskaya-19 mutant lines, along with the wild-type parent, were further used for SR phenotypic evaluation and for the molecular characterization and identification of new sources with effective *Lr* genes.

The phenotyping of mutant lines—focusing on SR to LR—was carried out using microscope examination of leaf assays inoculated by the aggressive *77WxR P. triticina* fungal isolate [3,11]. This examination showed that of the 42, 15, and 17 samples representing 300-, 350-, and 400-Gy-treated mutants, respectively, 37 (88.1%), 12 (80%), and 14 (82.4%) exhibited high APR to LR, and LR SR was observed after both treatment durations (72 and 168 h) (Figure 2). Thus, our field observations for APR were generally consistent with the microscope analyses, which were more informative and allowed the visualization of the pathogen structures. The microscope investigations of host–pathogen interactions in carriers of specific *Lr* genes revealed that among the analyzed mutant lines, *Lr1*, *Lr2a*, *Lr3a*, *Lr9*, *Lr24*, *Lr26*, *Lr29*, *Lr30*, *Lr34*, and *Lr38* NILs infected by isolate *77WxR* had the highest level of fungal haustoria development, although this was lower for *Lr3a*, *Lr26 Lr29* at the seedling stage (Figure 1). It has been reported that, except for *Lr47*, this isolate is virulent against *Lr* genes located in the A-genome, that is, *Lr10*, *Lr11*, *Lr20*, *Lr28*, *Lr37*, and *Lr49* [11]. Thus, the microscope analyses for HMC formation indicate the response difference between the parent (wild type) and mutant lines developed and that most of the mutant lines identified as APR to LR had a strong association with SR. Because of the low exposure to *P. triticina*, this gene can be used for the pyramiding of leaf-rust resistance [5]. The resistance conferred by this gene applied to triticale is reported for the first time in this study.

### 4.2. Hyperspectral Imaging System for the Quantification of Healthy and Diseased Plants Based on Different Indexes, and Discrimination between Susceptible and LR-Resistant Spring Wheat Mutant Lines

As shown in Figure 5 and Figure 6, the leaves that were infected by LR for two weeks exhibited some characteristic spectral responses across the spectrum. Infected leaves of wheat genotypes showed a relative reflectance increase in visible light, with the highest reflectance at 462 nm and 644 nm, while near-infrared reflectance peaked at 1936 nm and 2392 nm. These peaks were associated with changes (decrease) in chlorophyll content.

The increase in relative reflectance in visible light might be associated with a breakdown of the chloroplast and some visible foliar symptoms [77], whereas the peak near-infrared reflectance at 1936 nm and 2392 nm might be related to the loss in water content [78], which could also be observed in the WI and NDVI value differences between non-infected and infected plants (Table 1). Among the indexes used to investigate LR severity in this study, five spectral indexes—namely red edge NDVIs, SIPI, RVSI, WI, and NDWI—demonstrated significant potential for identifying LR disease at the seedling growth stage (Table 1). It has been reported that PhRI is highly correlated with wheat rust disease severity [79] and three-band PhRI2 in the early mid-growth stage [59]. According to our results, this index in two versions is less able to distinguish between healthy and infected wheat than the other five indexes mentioned above. The ARI has previously been utilized for estimating vegetation fraction [63] and detecting wheat yellow rust [59]. Our results also show that ARI can effectively identify wheat LR disease, which is consistent with previous research.

Spectral analysis of spring wheat leaves for the responses of LR-susceptible and -resistant mutant lines after LR infection indicated that the three most significant peaks—at 485.50, 694.57, and 1467.0 nm—clearly differentiate these genotypes (Figure 6 and Figure 7). It was previously shown that the band at the 705 nm region is an indicator of wheat LR [34]. Another major significant difference between LR-susceptible and -resistant lines is in the near-infrared region, which could be associated with WI and NDWI (Table 1). This finding facilitates the differentiation of the response between LR-susceptible and -resistant genotypes and can be considered critical for the discrimination of the degree of wheat resistance to LR.

### 4.3. Genotyping with KASP Markers and Comparison with Phenotyping Evaluation

Functional or gene-specific markers are the most valuable resources for genotyping, as they provide an excellent opportunity to effectively practice MAS [42]. InDels and SNPs are types of mutations in the exomic or regulatory regions of genes and can arise due to natural or induced mutation, resulting in multiple alleles [80,81]. KASP assay is a promising method of high-throughput SNP genotyping—which is currently the most competitive genotyping technology—and is being developed and validated for different traits in wheat [38,82]. This includes several KASP markers based on resistance genes, such as *Lr21* [83], *Lr16* [84], *Lr64* [84], and *Lr79* [79]. Here, we have demonstrated the effectiveness of newly developed KASP assays for genes conferring LR tolerance in spring wheat. These assays accelerate the development or progress of the deployment of rust disease tolerance-conferring genes in wheat improvement in a cost-effective manner. Moreover, the deployment of superior alleles in improved cultivars could be enhanced with the availability of efficient molecular diagnostics [40,41,42,43].

In the present work, different Gy-irradiation-dosed spring wheat mutant breeding lines were validated for SNPs in nine *Lr* genes using KASP markers (*Lr1*, A/G, *Lr2a*, T/C, *Lr3*, *Lr9*, *Lr10*, C/T, *Lr14*, *Lr17*, A/G, *Lr19*, T/C, and *Lr24*). Among them, three KASP markers for genes *Lr14*, *Lr19*, and *Lr24* were monomorphic and thus excluded from the analysis. The genotyping results for the other six *Lr* genes—*Lr1*, *Lr2a*, *Lr3*, *Lr9*, *Lr10*, and *Lr17*—are shown in Figure S1 and summarized in Table 2. The 14 mutant lines generated from 17 400-Gy-dosed lines (82.4%) were classified as homozygous for “*a*” alleles in six studied *Lr* genes and showed no signs of fungal haustoria development.

In the 16 mutant lines generated through the 350-Gy dosage of irradiation, a similar picture of KASP allele distribution as to that for the 400-Gy-dosed lines was found, in that 13 genotypes (81.3%) were homozygous for SNP genotyping for the “*a*” alleles of the *Lr* genes studied in this work. Favorable allelic presence in the given genes was associated with high LR resistance, with the phenotypic appearance of low or no fungal haustoria production.

Genotyping with KASP markers for *Lr1*, *Lr2a*, *Lr3*, *Lr9*, *Lr10*, and *Lr17* applied to the mutant breeding lines generated via 300-Gy irradiation classified 39 genotypes (92.86%), except line MK/84, as homozygous for “*a*” alleles in six given *Lr* genes, and they showed no symptoms of fungal haustoria development (Supplement Figure S1, and summarized in Table 4). The other three 300-Gy-developed mutant lines, except for line MK/30, with “*b*” alleles in the studied *Lr* genes, were associated with the LR-susceptible response.

Thus, KASP markers for *Lr1*, *Lr2a*, *Lr3*, *Lr9*, *Lr10*, and *Lr17* tested on various Gy-dosed spring wheat mutant lines clearly indicate that the presence of “*a*” alleles in the given genes underpins LR tolerance and effectively differentiate resistant and susceptible genotypes. Interestingly, the combination of “*a*” and “*b*” alleles in *Lr3* and *Lr9*—and *Lr1*, *Lr2a*, *Lr10*, and *Lr17*—revealed in the 300-Gy-generated mutant line MK/54 was also favorable for the development of LR tolerance.

The stacking (or pyramiding) of multiple resistance genes is necessary to prolong resistance durability and enhance the effective use of *Lr* genes [1]. The *Lr* locus *Lr1* was mapped at the 5D [75,76], 5DL [77] chromosome of bread wheat and has been shown to likely continue to play a role in gene combination; it is also highly effective against avirulent pathotypes. This gene was also isolated so that the sequence could be used to validate the linkage to our markers used in this study (Table 2). *Lr2* is a complex locus comprising at least three resistance alleles, of which *Lr2s* is the most important because it confers the widest array of resistance and the lowest infection types. According to our results, *Lr2* could be a useful component of multiple-gene resistance and cannot be molecularly separated from other *Lr2* resistances. This suggests that LR isolate *77WxR* and the SNP markers are not suitable for differentiating *Lr2a*, *Lr2b*, and *Lr2c*.

Three resistance alleles have been described at or near the *Lr3* locus; these are widely dispersed and probably have a minor role in gene combinations. *Lr9* is present on chromosome 6B [84] and was transferred to Chinese Spring wheat from *Triticum umbellulatum*. Translocation of *Lr9* has not been widely deployed despite its widespread effectiveness.

In conclusion, the new spring wheat LR mutant lines developed in this study possess a combination of resistance *Lr* genes that would be useful to incorporate into future studies. The allele “*a*” associated with high ADP and SR to LR was detected in a number of mutant lines developed here. The high LR resistance response of mutant lines was highly correlated with the predicted combination of *Lr2a* and *Lr3a*. In our study, three resistance alleles were found near the *Lr3* locus. Such clusters of genes are seen in a variety of fungal species, and they are conserved throughout eukaryotes as described in papers published previously [84].

## Figures and Tables

**Table 1:** Virulence/avirulence pattern of leaf rust isolates detected at the seedling stage of near-isogenic wheat lines carrying *Lr* genes singly. In general, virulence was observed against *Lr1*, *Lr2a*, *Lr2b*, *Lr2c*, *Lr11*, *Lr12*, *Lr13*, *Lr14a*, *Lr14b*, *Lr15*, *Lr16*, *Lr17*, *Lr18*, *Lr20*, *Lr21*, *Lr22a*, *Lr22b*, *Lr23*, *Lr30*, *Lr32*, *Lr35*, *Lr36*, *Lr37*, and *LrB*, and avirulence against *Lr9*, *Lr19*, *Lr24*, *Lr25*, *Lr29*, *Lr45*, *Lr51m*, *Lr53*, and *LrTm*.

Isolate	Virulence	Avirulence
4083	*Lr3a*, *Lr3bg*, *Lr3ka*, *Lr10*, *Lr17b*, *Lr33*, *Lr40*, *Lr46*, *Lr49*, *Lr50*, *Lr52*	*Lr4*, *Lr26*, *Lr27*, *Lr28*, *Lr34*, *Lr39*, *Lr47*, *Lr48*,
4171	*Lr15*, *Lr16*, *Lr27*, *Lr32*, *Lr33*, *Lr39*, *Lr40*, *Lr46*, *Lr48*, *Lr49*, *Lr50*,	*Lr3a*, *Lr3bg*, *Lr3ka*, *Lr4*, *Lr10*, *Lr17b*, *Lr26*, *Lr28*, *Lr30*, *Lr34*, *Lr47*, *Lr51*, *Lr52*, *Lr53*, *LrTm*
*77WxR*	*Lr2c;B*, *Lr3a*, *Lr3bg*, *Lr3ka*, *Lr4*, *Lr10*, *Lr17b*, *Lr26*, *Lr28*, *Lr30*, *Lr32*, *Lr33*, *Lr39*, *Lr40*, *Lr44*, *Lr48*, *Lr49*, *Lr52*	*Lr27*, *Lr34*, *Lr40*, *Lr43*, *Lr46*, *Lr47*, *Lr50*,
FI17	*Lr2c;B*, *Lr10*, *Lr11*, *Lr12*, *Lr13*, *Lr14a*, *Lr14b*, *Lr17b*, *Lr18*, *Lr26*, *Lr27*, *Lr28*, *Lr30*, *Lr32*, *Lr33*, *Lr34*, *Lr39*, *Lr40*, *Lr44*, *Lr46*, *Lr48*, *Lr49*, *Lr50*, *Lr52*	*Lr3a*, *Lr3bg*, *Lr3ka*, *Lr4*, *Lr38*, *Lr41*, *Lr43*, *Lr47*
HkLr13	*Lr2c;B*, *Lr3a*, *Lr3ka*, *Lr10*, *Lr11*, *Lr12*, *Lr13*, *Lr14a*, *Lr14b*, *Lr17b*, *Lr23*, *Lr27*, *Lr30*, *Lr32*, *Lr44*, *Lr46*, *Lr50*, *Lr52*	*Lr3bg*, *Lr4*, *Lr26*, *Lr28*, *Lr33*, *Lr34*, *Lr38*, *Lr39*, *Lr40*, *Lr41*, *Lr43*, *Lr47*, *Lr48*, *Lr49*
Genotypes (NILs, accessions) carrying *Lr* genes singly	*Lr1*, *Lr2a*, *Lr2b*, *Lr2c*, *Lr2c;B*, *Lr3a*, *Lr3bg*, *Lr3ka*, *Lr4*, *Lr9*, *Lr10*, *Lr11*, *Lr12*, *Lr13*, *Lr14a*, *Lr14b*, *Lr15*, *Lr16*, *Lr17*, *Lr17b*, *Lr18*, *Lr19*, *Lr20*, *Lr21*, *Lr22a*, *Lr22b*, *Lr23*, *Lr24*, *Lr25*, *Lr26*, *Lr27*, *Lr28*, *Lr29*, *Lr30*, *Lr32*, *Lr33*, *Lr34*, *Lr38*, *Lr39*, *Lr40*, *Lr41*, *Lr43*, *Lr44*, *Lr45*, *Lr46*, *Lr47*, *Lr48*, *Lr49*, *Lr50*, *Lr51*, *Lr52*, *Lr53*, *LrB*, *LrTm*	

**Table 2:** Spectral indexes used to investigate LR severity caused by *P. triticina* fungal isolate *77WxR* inoculation of spring wheat seedlings in this study.

Spectral Index	Definition	Formula	Index		Index Ratio between Non-Infected/Infected Plants	References
			Non-Infected Plants	LR-Infected Plants		
NDVI	Normalized difference vegetation index	(R_800_ − R_680_)/(R_800_ + R_680_)	0.444 ± 0.040	0.407 ± 0.054	1.09	[32]
GNDVI	Green normalized difference vegetation index	(R_800_ − R_550_)/(R_800_ + R_550_)	0.595 ± 0.03	0.547 ± 0.11	1.09	[18]
PRI	Photochemical/ physiological reflectance index	(R_570_ − R_531_)/(R_570_ + R_531_)	0.127 ± 0.00	0.129 ± 0.007	0.98	[57]
RNDVI	Red edge normalized difference vegetation index	(R_740_ − R_690_)/(R_740_ + R_690_)	0.368 ± 0.058 **	0.289 ± 0.028	1.27	[32]
PhRI1	Physiological reflectance index 1	(R_550_ − R_531_)/(R_550_ + R_531_)	0.085 ± 0.007	0.089 ± 0.003	0.96	[58]
PhRI2	Physiological reflectance index 2	(R_570_ − R_525)_/(R_570_ + R_700_)	0.1067 ± 0.0038 *	0.1006 ± 0.003	1.06	[59]
MCARI	Modified chlorophyll absorption in reflectance	[(R_700_ − R_670_) − 0.2·(R_700_ − R_500_)] · (R_700_/R_670_)	0.216 ± 0.03	0.208 ± 0.03	1.04	[18]
SIPI	Structure insensitive pigment index	(R_800_ − R_445_)/(R_800_ − R_680_)	1.572 ± 0.16 **	0.912 ± 0.01	1.72	[60]
MTCI	Medium-resolution imaging spectrometer (MERIS) terrestrial chlorophyll index (MTCI)	(R_750_ − R_710_)/(R_710_ − R_680_)	2.293 ± 0.64	1.933 ± 0.63	1.19	[61]
RVSI	Ratio vegetation index	(R_712_ + R_752_/2) − R_732_	6599.3 ± 1161.5 **	4844.35 ± 826.0	1.36	[62]
ARI1	Anthocyanin reflectance index 1	(R_550_)^−1^ − (R_700_)^−1^	−543.78 ± 108.5 **	−411.29 ± 93.6	0.76	[63]
ARI2	Anthocyanin reflectance index 2	1/(R_860_ − R_790_) − 1/(R_790_ − R_750_)	−0.00005 **	−0.00008	0.63	[59]
	Leaf carotenoid content, water index, and LR disease severity index					
CRI	Carotenoid reflectance index	(1/R_510_) − (1/R_550_)	0.000076 *	0.000106	0.72	[60]
WI	Water index	R_900_/R_970_	1.068 ± 0.053 *	0.858 ± 0.014	1.24	[57]
NDWI	Normalized difference water index	(R_860_ − R_1240_)/(R_860_ + R_1240_)	0.144 ± 0.042 **	−0.064 ± 0.020	−2.24	[64]
LRDSI	LR disease severity index	6.9 × (R_605_/R_455_) − 1.2	15.74 ± 1.11 **	21.21 ± 3.47	0.74	[34]

R is the reflectance value at wavelength λ. * significant difference (*p* < 0.01), ** significant difference (*p* < 0.05).

**Table 3:** Selected markers for the detection of *Lr*-gene-linked SNPs. Based on the information from NILs, the closest markers—which could be validated for differentiation between *Lr*-gene-carrying NILs and those without *Lr* genes—are shown in bold letters and were used for further analysis within the study. Markers could not differentiate between *Lr2a*, *Lr2b*, and *Lr2c*, and *Lr17* and *Lr37* (**). Physical positions of the SNP markers were identified based on information provided by Sun et al. [67,61]. Physical regions of cloned genes were identified based on a reference genome of Chinese spring wheat.

*Lr* Gene	CChr.	Markers on Chromosome	SNPs	Region (Flanking Marker Lowest bp)	Region (Flanking Marker Highest bp)	Marker for Selection Linked to Resistance and Selected for KASP Analysis	R Allele (aa)	S Allele (bb)	Position of Cloned Gene (Start)
*Lr1*	5D	2636	14	560,263,603	561,797,031	wsnp_Ex_c11055_17927692	T	C	568,850,072
*Lr3a*	6B	1743	88	705,159,008	720,529,223	wsnp_Ex_rep_c69373_68312188	C	T	n.d.
*Lr9*	6B	1655	17	709,532,733	712,243,672	wsnp_Ex_c54772_57527387	T	C	n.d.
*Lr10*	1A	1967	20	8,296,948	12,505,407	wsnp_Ku_c183_358844	A	G	12,230,495
*Lr14a*	7B	2097	16	734,296,333	750,589,139	wsnp_Ex_c32905_41484291	C	T	n.d.
*Lr17* **	2A	2637	31	259,237	12,001,189	wsnp_Ex_c19516_28483751	C	A	n.d.
*Lr19*	7D	2368	182	370,181,480	636,770,931	wsnp_Ex_c5884_10325223	T	C	n.d.
*Lr24*	3D	2173	176	511,657,120	614,691,597	wsnp_Ku_c7264_12545135	C	T	n.d.

**Table 4:** Summary evaluation results for resistant (R) and sensitive (S) phenotypes of haustorium mother cells (HMCs), and genotyping of six *Lr* genes (*Lr1*, *Lr2a*, *Lr3*, *Lr9*, *Lr10*, and *Lr17*) for four-day-old seedlings of 75 mutant breeding lines infected by fungal *P*. *triticina* isolate *77WxR*. The mutant lines were generated using a range of 300-, 350-, and 400-Gy irradiation and compared with the wild parent control, spring wheat cv. Kazakhstanskaya-19. Full results are presented in Supplement Figure S1.

X-ray Treatment, Gy	Phenotyping for HMCs	Genotyping of Mutant Lines	
		*aa*	*Bb/Failed*
400	Not developing (R)	14	0
	Highly developed (S)	0	3 ^a^
350	Not developing (R)	13	0
	Highly developed (S)	0	3 ^b^
300	Not developing (R)	38 ^c^	1
	Highly developed (S)	1	2 ^d^
Total		66	9
*Control*, *WT*	Not developing (R)	1	0

Notes: ^a^ Two lines had alleles “*a*” in *Lr9* and *Lr10*; ^b^ One line had allele “*a*” in *Lr10*; ^c^ Allele “*b*” was found in *Lr3* in one line, and in four other *Lr* genes in another line; ^d^ One line had allele “*a*” in *Lr10*.

## Data Availability

Data produced in this study are presented in this paper and in the Supplementary Materials.

## Supplementary Materials

The following supporting information can be downloaded at: https://www.mdpi.com/article/10.3390/cimb46010045/s1. Supplement S1: Genomic DNA Miniprep Method. Supplement Figure S1: Scatter plot for selected KASP assays showing clustering of genotypes on the Y- and X-axes.
