# Supplementary material for: Phenotyping and Exploitation of Kompetitive Allele-Specific PCR Assays for Genes Underpinning Leaf Rust Resistance in New Spring Wheat Mutant Lines
Source: Curr Issues Mol Biol. 2024 Jan 12;46(1):689–709. doi: 10.3390/cimb46010045 (PMC10814123; doi:10.3390/cimb46010045)
Supplement: Supplementary file 1 [file cimb-46-00045-s001.zip › cimb-2772528-Figure S1.pdf]

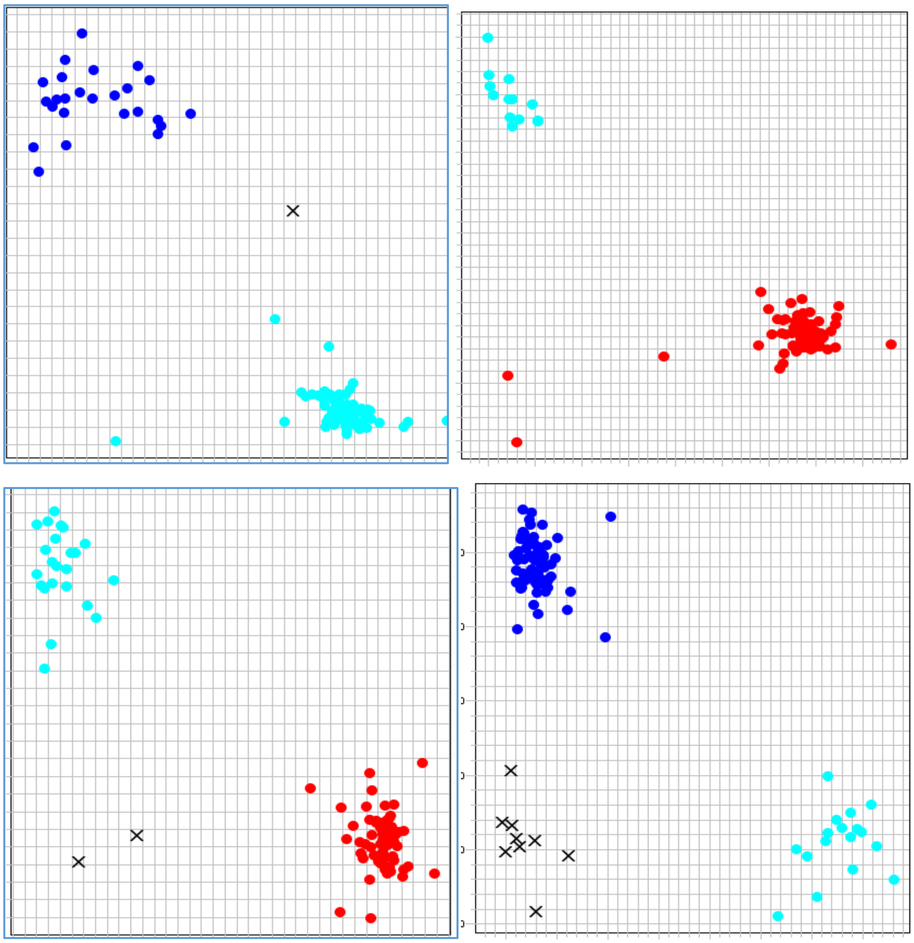

Supplement Figure S1. Scatter plot for selected KASP assays showing clustering of genotypes on the Y- and X-axes. Genotypes colored red have a HEX-type allele; genotypes colored blue have a FAM-type allele. Top left and right corners, KASP assays for *Lr1*–A/G, *Lr2* –T/C, respectively. Bottom left and right corner, KASP assays for *Lr10* – C/T and *Lr14a* –C/T, respectively.
