# Supplementary material for: Phenotyping and Exploitation of Kompetitive Allele-Specific PCR Assays for Genes Underpinning Leaf Rust Resistance in New Spring Wheat Mutant Lines
Source: Curr Issues Mol Biol. 2024 Jan 12;46(1):689–709. doi: 10.3390/cimb46010045 (PMC10814123; doi:10.3390/cimb46010045)
Supplement: Supplementary file 1 [file cimb-46-00045-s001.zip › cimb-2772528-supplement S1.pdf]

Genomic DNA Miniprep Method

note: (o.n. 4°C) = steps indicated like this can be extended overnight without negative impact on the results

1 place 300 mg of fresh leaf material into plastic bag, add 1.2 ml of **2*CTAB-buffer** add 2% of β-Mercaptoethanol just before use, seal the bag and macerate.

(o.n. 4°C)

2 incubate bags containing macerated leafs for at least 60 min at 65°C (can be extended for several hours)

3 cut the edge of the bag and empty the solution (~900 µl) into a 2.2 ml microfuge tube

4 add 800 µl **Chloroform:Isoamylalcohol** (24:1)

(o.n. 4°C)

5 place the tubes into rack and then into overhead-shaker (half-speed) for 15 min
(after shaking, o.n. 4°C)

6 centrifuge for 15 min at 10‘000 x g (13'000 rpm, Heraeus Biofuge pico)

7 take 800 µl of the supernatant and transfer into 1.5 ml microfuge tube

8 add 5 µl **RNase A solution**, mix by inverting the tubes, incubate for 15 min at 37°C

9 precipitate the DNA by adding 560 µl **Isopropanol** (0.7 Vol !) and inverting the tube until well mixed (do this at room temperature to prevent extensive precipitation of carbohydrates)

10 to pellet the DNA centrifuge for 10 min at 10'000 x g (13'000 rpm, Heraeus Biofuge pico) at 4°C (a white pellet should be clearly visible at this stage)

11 remove the supernatant by aspiration (use disposable yellow tip attached to a vacuum line; be careful, the pellet is only loosely attached to the wall of the tube!)

12 add 1 ml of **Wash solution I** and incubate for 15 min

(o.n. 4°C)

13 aspirate the supernatant carefully (see step 11) and replace by 1 ml of **Wash solution II**

14 short incubation (~ 5 min, if pellet remains for a too long period of time in wash II it becomes translucent and is difficult to see while aspirating wash II) and aspiration of **Wash solution II**

15 short spin and aspiration of the remaining traces of **Wash solution II**

16 dry the pellet 5-10 min at room temperature (or 1 min in speedvac). If pellet is overdried dissolving of the DNA is difficult and takes some time.

17 dissolve the pellet in 80-100 µl **TE-buffer** (pH 8.0), measure DNA concentration by UV-absorption at A= 260 nm. (If many samples are going to be processed estimate DNA conc. of some samples and dissolve the pellets in the following in an appropriate volume to end up with a working-DNA-solution and to avoid further estimation of DNA conc and dilution steps)

Solutions:

2*CTAB

2% CTAB (Hexadecyltrimethylammoniumbromid, F 52369, FLUKA AG, Switzerland)	20 g
200 mM Tris/HCl (pH 8.0)	200 ml of 1 M stock
20 mM EDTA	40 ml of 0.5 M stock
1.4 M NaCl	81 g
1.0% PVP (Polyvinylpyrrolidone K30)	10 g

adjust to 1 l with sterile ddH_2O, sterile filtrate solution,
add 20 ml of BME just before use 0.28 M β-Mercaptoethanol (BME) (Mr=78.13; d=1.115)

RNase A [10 mg/ml]

RNase A (Fluka 83832)	200 mg
10 mM sodium acetate pH 5.2	60 µl 3 M stock
100 mM Tris/HCl pH 7.4	2 ml of 1 M stock

dissolve Rnase A in 18 ml 0.01 mM NaAc and boil in water bath for 30 min, cool down slowly to room temperature, add 0.1 vol 1M Tris/HCL, check remaining DNase activity and store in aliquots at –20°C

Wash solution I

76% Ethanol	760 ml of 100% Ethanol
200 mM Sodium acetate (anhydrous)	66.7 ml of 3 M NaAc
	adjust to 1 l with sterile ddH_2O

Wash solution II

76% Ethanol	760 ml of 100% Ethanol
10 mM Ammonium acetate	771 mg NH_4Ac
	adjust to 1 l with sterile ddH_2O

TE-buffer

10 mM Tris/HCl pH 8.0	10 ml of 1 M stock
1 mM EDTA	2 ml of 0.5 M stock
	adjust to 1 l with sterile ddH_2O

Literature:

Stein N., G. Herren and B. Keller (2001) A new DNA extraction method for high-throughput marker analysis in a large genome species like */Triticum aestivum/* L. Plant Breeding 120, 354-356.
